# Context and the human microbiome

**DOI:** 10.1186/s40168-015-0117-2

**Published:** 2015-11-04

**Authors:** Daniel McDonald, Amanda Birmingham, Rob Knight

**Affiliations:** Department of Computer Science, University of Colorado at Boulder, Boulder, CO 80304 USA; BioFrontiers Institute, University of Colorado, 3415 Colorado Avenue, Boulder, CO 80304 USA; Center for Computational Biology & Bioinformatics, Department of Medicine, University of California San Diego, 9500 Gilman Drive, La Jolla, CA 92093 USA; Department of Pediatrics, University of California San Diego, 9500 Gilman Drive, La Jolla, CA 92093 USA; Department of Computer Science and Engineering, University of California San Diego, 9500 Gilman Drive, La Jolla, CA 92093 USA

**Keywords:** Microbiome, American Gut Project, Reference database, Meta-analysis

## Abstract

Human microbiome reference datasets provide epidemiological context for researchers, enabling them to uncover new insights into their own data through meta-analyses. In addition, large and comprehensive reference sets offer a means to develop or test hypotheses and can pave the way for addressing practical study design considerations such as sample size decisions. We discuss the importance of reference sets in human microbiome research, limitations of existing resources, technical challenges to employing reference sets, examples of their usage, and contributions of the American Gut Project to the development of a comprehensive reference set. Through engaging the general public, the American Gut Project aims to address many of the issues present in existing reference resources, characterizing health and disease, lifestyle, and dietary choices of the participants while extending its efforts globally through international collaborations.

## Review

### Background

In the last few years, the study of the bacteria, archaea, microbial eukaryotes, and viruses that inhabit the human body (particularly the large intestine) has revealed a remarkable biological and functional diversity [1–6]. These organisms, collectively known as the microbiome, potentially outnumber human cells in 10:1 [7] and vastly expand on the functional capabilities provided by our genomes. Disruption in these microbial communities, also known as dysbiosis, has been causatively associated by transferring microbiomes and phenotypes to mice associated with human Kwashiorkor [8] (a wasting disease endemic to Africa) and obesity [9]. Numerous correlative associations in humans and mouse models have also been observed in a broad spectrum of complex diseases including autism spectrum disorder [10], inflammatory bowel disease [11], type 2 diabetes [12], colorectal cancer [13], depression [14] (see [15] for a detailed review on the brain-gut-microbe axis), and more.

The implication of the microbiome in human health is immense, with prospects for novel medical products including therapeutics and clinical assays. This has led to large investments in both academia [3] and industry [16]. Although such research could have a profound impact on human society both in first- and third-world countries, we are just scratching the surface of understanding the complexity of this vital organ. As such, identifying means that improve the pace of research is arguably a matter of human health on a global scale.

A crucial and missing component of microbiome research is a robust and comprehensive reference set of microbiome samples and metadata about those samples that are available for public, unrestricted use. Such a dataset would characterize what we know about diversity of the human microbiome and its relationship to the health and lifestyle choices of individuals, providing much-needed context against which to compare findings of focused studies such as those on particular disease populations. This reference would allow researchers to place their study in the framework of what is already known in order to better interpret observed patterns (compelling examples of this can be found in [17, 18]). It would also enable stringent hypothesis testing and evaluation of effect sizes. A robust reference dataset must be built on top of a cross-sectional study design in order to understand the variation in the population, while also including rich longitudinal components to enable an understanding of how species structure changes over time.

In this review, we highlight the importance of reference sets in human microbiome research, limitations of existing resources, technical challenges to employing reference sets, examples of prototypical reference usages, and contributions of the American Gut Project to addressing some of these issues. Discussion will focus on the 16S ribosomal RNA (16S rRNA) gene, which is a popular locus for use in microbiome studies over a wide range of environment types [19–23] and is the core locus assayed in the American Gut Project. Construction of references based on other loci is important for studying microbial eukaryotes, viruses, and interactions between these organisms, but high-throughput study of these other components of the community is not yet cost-effective.

### Importance of reference sets in human microbiome research

The community structure of the human microbiome is the result of a multifactorial process that involves succession over time [24], is influenced by host genetics [25], and is affected by lifestyle choices [26, 27]. Communities are made up of thousands of microbial species, with the predominant microbial biomass residing in the human large intestine. Fascinatingly, within the human gastrointestinal tract, it appears that multiple organisms are capable of fulfilling common ecological niches, leading to remarkably different microbial communities that possess similar functional potential [3]. Furthermore, while variations in the human genome are minute across the population, variations in the human microbiome on geographical and temporal scales are immense [28, 29]. Despite investments of hundreds of millions of dollars, we still do not understand the distribution of community structures in healthy individuals [30], but we do know that when studies of the microbiome are performed without a concern for integration with existing studies, effects of significant biological importance can be easily missed [31].

A well-characterized reference dataset can be used to test hypotheses and, conversely, to derive testable hypotheses from the reference itself. For instance, inflammatory bowel disease has been observed to be associated with a microbial dysbiosis index (MD-index) that is the ratio of the relative abundances of a set of pro-inflammatory taxa to a set of anti-inflammatory taxa [32]; a robust reference set would allow assessment of the hypothesis that diet or lifestyle factors are strongly correlated to this index within the general public as well. In an opposite example, a significant correlation between diversity and time of the year was observed in the American Gut reference set [33]. Because it appears that individuals have a higher diversity during the holiday season in the US, one might hypothesize that it is the holidays and not the time of the year that drives the correlation—possibly due to changes in exercise and diet patterns. This putative effect can then be tested once the project acquires sufficient samples from western countries in the southern hemisphere.

A comprehensive reference dataset will also help researchers make rational decisions about sample size by enabling power calculations, which can greatly impact the utility of a study [34]. Such a dataset is also crucially necessary to support characterization of the effect sizes of variables (e.g., antibiotic use). Within the microbiome field, effect size for many variables of interest is not yet well understood, and many that are important in diseases with complex etiologies such as autism [10] are likely to be small. Well-characterized references offer the possibility for a researcher to expand their dataset by pulling reference samples to augment their own [29], particularly when meta-analysis (i.e., combination of summarized data from multiple studies) is taken into consideration during the design phase for a study.

### Limitations of existing reference sets

The $173 million NIH-initiated Human Microbiome Project (HMP) set out to characterize the human microbiome at a population scale and to define standard reference datasets to be used for human microbiome research [35]. The resulting 16S rRNA datasets are composed of samples from 242 individuals, all of whom were medical students in the USA and were certified healthy by medical professionals. Thousands of samples were collected from these individuals at one to three time points, covering 15 to 18 sampling sites depending on the sex of the individual. These samples were evaluated using two different regions of the 16S gene (leading to two distinct datasets—V1-3 and V3-5) [31] and were processed at four different sequencing centers. Phenotypic information about the individuals was collected, but while the sequence data associated with the samples are publically available, access to any de-identified information about the individuals requires rigorous approval mechanisms.

Although the HMP generated an incredible volume of data, numerous design, technical, and access decisions affecting the HMP dataset have made reuse challenging. For instance, the decision to sample a few people extensively rather than a large number of people minimally (i.e., a cross-sectional study design) led to observation of only a small fraction of the diversity present with the population [28] and resulted in small sample sizes for different stratifications in the dataset [36], effectively removing the potential to observe demographic or regional differences. The choice to sequence multiple loci within the 16S rRNA gene resulted in data that are impractical to combine due to technical bias as amplification performance differs between primers [31, 37]. Furthermore, because the study design was not sufficient to elucidate the effect of employing multiple sequencing centers (which has been observed in other contexts; see the Microbiome Quality Control Project (MBQC) [38]), this issue must still be actively evaluated to assess the potential for technical biases. Host information, such as age and sex, are nearly prohibitive to access, requiring dean-level signatures for each individual piece of metadata, which makes explaining any systematic patterns in the data impossible without knowing in advance what pattern one expects to see. The end result is that use of the HMP 16S rRNA as a robust reference set has proven difficult.

In contrast to the HMP, the Global Gut project [28] set out to characterize microbial diversity at spatial and temporal scales. To do this, the researchers collected samples from three distinct populations (US citizens, Malawians, and Venezuelan Amerindians), the latter two of which are culturally distinct from western populations. Within each population, samples were collected cross-sectionally over an age gradient. Notably, the two non-western populations appear to be completely distinct from the western individuals, suggesting the limited population size and emphasis of the HMP grossly underestimate the variation in community structure across the human race. However, the populations do intersect on samples collected from infants, suggesting that it is potentially lifestyle, diet, or environmental choices that shape our microbiomes as we age (including interaction with our genetic predisposition [25]). Although the sequence data are readily available for reuse, the distribution of many of the study variables is not approved, limiting the long-term usefulness of the samples. (It should be noted that the Global Gut did not intend to be a reference for microbiome research, but the populations represented in the dataset are extremely difficult to collect samples from and have shown to be useful in adding perspective for independent projects [29, 39]).

Lack of access to the full set of metadata variables associated with these earlier studies is crippling, as interpretation of the observational data can only happen within the context of the collected variables. From a practical standpoint, if a systematic pattern is observed in the data, but there are not any variables that explain the pattern, then the researcher cannot support a hypothesis about the pattern without collecting new information (which may be impractical and impossible or introduce recall bias). Similarly, confidence in the face of confounding variables is reduced if only a limited number of variables are tracked. As a concrete example, if researchers broadly characterize subjects by diet type (e.g., vegan) and observe an effect, the researchers will be unable to assess whether the effect is due to the diet type itself, differential fiber consumption, protein source, etc., unless these other variables are recorded. Given that researchers typically do not know the answer in advance of a study, it is imperative that study designs strive to collect as much information as feasible.

### Technical challenges to employing reference sets

Even a well-designed and carefully collected reference must be employed with caution in order to minimize spurious variation and contain necessary computational effort. The first of these needs arises since reference-based analyses assume that any systematic compositional differences inherent in the data outweigh any technical variation, which is particularly problematic when combining data generated from different protocols or platforms [31]. In fact, biological conclusions can be driven by technical variation even if the researchers are careful (as in [40], where samples were found to cluster by the extraction kit used), which underscores the need for accepted community standards for sample handling, sequencing, and data analysis in order to minimize the potential for introducing such variation. Bioinformatic strategies to mitigate any remaining variation, such as trimming sequences to a common length between studies, have shown to help normalize platform bias [29]. Sometimes, stronger measures are necessary: for example, the American Gut Project received samples from self-reported healthy individuals that contained levels of gammaproteobacteria beyond anything previously observed in healthy populations (although similar to those observed in samples from ICU patients [*manuscript in prep*]). It was determined that these blooms likely stemmed from the shipping conditions for some samples. The blooms can be bioinformatically subtracted from the dataset [*manuscript in prep*] by removing organisms observed to bloom (as has been observed to happen in storage [*unpublished observations*]). As a result, any meta-analysis that leverages the American Gut data must perform this same subtraction in order to equalize bias that the filter introduces. Ongoing studies of stability [41, 42] are explicitly exploring the effect of different types of storage effects so that they can be controlled for as necessary in the future.

Once technical variation has been minimized, the comparative analysis can begin. Many researchers, particularly those at remote sites, do not have access to large-scale compute instruments and must rely on commodity hardware for data analysis; this creates the temptation to employ analysis techniques that require as little computation as possible. However, some such techniques are particularly vulnerable to artifacts caused by combining dissimilar datasets.

An exemplar of this issue is the assignment of operational taxonomic units (OTUs). The primary data type used in analysis of a microbiome study is the OTU table [43, 44], a matrix in which the rows represent observations (OTUs), the columns represent samples, and the elements correspond to the number of counts of a given observation within a sample. In order to be comparable, a reference and a study must have their sequence data assigned to a shared set of OTUs (i.e., partitioned into a common set of bins). OTUs themselves are clusters of similar sequences, with the similarity threshold generally set at 97 % by sequence identity, and are typically determined in one of three ways as summarized in Table 1 (for a comprehensive review of OTU picking, please see [45]; each of these methods is named in terms of its OTU reference, but nota bene that this represents a distinct concept from that of the reference datasets discussed throughout). The first is a closed-reference approach in which all the sequence data for the input study and the microbiome reference set are compared against a curated 16S rRNA database such as Greengenes [46] to identify which known OTUs are represented. This is computationally tractable even for very large studies since the evaluation of every sequence is independent of every other and since the reference dataset’s OTU assignments can be computed just once (and in advance). The second strategy, known as de novo picking, defines novel OTUs based on the sequences in a study. This is computationally expensive, as all the data must be maintained in memory in order to determine the clusters, and the process is very complex to parallelize. The third approach, open-reference picking, is a hybrid method in which sequences are first compared to a database of known OTUs as described above, after which those that fail to match to a known OTU are then put through a de novo step.

**Table 1:** A comparison of OTU-picking strategies

Strategy	Pros	Cons	Data combination bias
Closed-reference	• Is extremely parallelizable	• Is limited to finding diversity present in OTU reference	• May show large bias if combining studies with differential representation in the reference
	• Computes reference assignments only once		
	• Is highly unlikely to retain non-16S sequences		
	• Supports and reads fragments from multiple loci		
	• Gets the phylogeny and taxonomy for free		
De novo	• Utilizes all of the sequences	• Must hold all sequence data in memory	• May generate spurious OTUs if combining studies with differential error profiles
	• Requires no OTU database	• Is very complex to parallelize	
	• Can group organisms distinct from anything seen before	• Produces spurious OTUs without pre-filtering	
	• May produce phylogenies sensitive to subtle differences in OTUs	• Is infeasible if data are from multiple loci	
		• Must redo OTU picking with all data being combined	
Open-reference	• Leverages an OTU database but also utilizes sequences that do not match to that database	• Produces spurious OTUs without pre-filtering	• Shows less bias due to differential diversity representation than closed-reference
		• Is infeasible if data are from multiple loci	
	• Is modestly parallelizable	• Must redo OTU picking with all data being combined	
			• Shows less bias due to differential error profiles than de novo

Studies employing a reference set typically rely on the closed-reference approach to minimize compute since only the input study need be evaluated and can be done so in an embarrassingly parallel fashion. Another benefit is that the closed-reference strategy is unlikely to result in OTUs composed of non-16S sequence, as the reference is expected to only contain 16S exemplars; furthermore, comprehensive references like Greengenes typically contain only near-full-length reads, thus allowing researchers to combine data represented by multiple variable regions. Of course, any annotation information about the reference, such as the phylogenetic relationship between the data contained or annotations such as taxonomy, can be attached to the input study data “for free.” Unfortunately, this strategy can only classify sequences that are reasonably similar to those in the reference database. Combining studies with differential representation in the reference (e.g., samples from different environments) can lead to statistically significant patterns in the data that are not driven by the underlying biology. As an example, imagine three samples A, B, and C where A is composed of *Escherichia coli* and both B and C are composed of *Escherichia coli* coli and *Bacillus subtilis*. If the reference is only composed of *Escherichia coli*, then all three samples will appear to be quite similar. However, if the reference includes *Bacillus subtilis*, then the conclusion drawn is quite different as A would be less similar to B and C.

In contrast to closed-reference, a de novo approach consumes more computational resources but requires no pre-existing reference and allows a researcher to assign OTUs to as much of the data as possible, including OTUs never before observed. It is capable of producing phylogenies sensitive to subtle differences in OTUs (as the representative member for an OTU is an actual study sequence), but this very sensitivity means that contamination in the data (e.g., non-16S sequence such as phiX) will also be clustered into OTUs unless the contamination is explicitly filtered out prior to OTU picking and that the method is not suitable for data drawn from multiple variable regions (as highlighted in [31]). Additionally, the distinct error profiles of the studies being combined (which can stem from the 16S protocol, variation in the master PCR mix, error profiles of the sequencing instrument used, etc.) may lead to spurious, study-specific OTUs (for example, the GC bias of the Illumina platform can lead to sequences that contain more GC than another platform which can result in OTUs specific to the platform even if the biological origin of the amplicon is the same). As a result, a meta-analysis that uses a de novo strategy must redo OTU picking after combining the sequence data from the studies.

The hybrid open-reference method steers a middle course: Since data that are not represented in the reference are recovered, bias driven by differential representation in the reference is reduced. In addition, since the amount of data being fed into the de novo step is minimized, the impact of study-specific error profiles is diminished. Open-reference OTU picking is modestly parallelizable and can be augmented with techniques such as use of a random subsample when constructing the intermediate de novo reference in order to accelerate its performance (details on the procedure can be found in [45] but are generally handled in by software without end-user intervention). However, open-reference picking shares a number of drawbacks with the de novo strategy, including necessity for pre-filtering, unsuitability for data from multiple variable regions, and necessity for re-picking when combining studies together. Of course, given the continued expansion of computational resources, sequencing throughput, and completed genomes available, optimal strategies for overcoming technical hurdles and enabling meta-analysis will require ongoing re-assessment.

### Examples of reference set usage

One of the first studies to combine multiple microbiome datasets (which these researchers are aware of) was the work by Lozupone and Knight [47], which aggregated sequence data from hundreds of studies in order to determine environmental factor(s) that explained the observed differences in microbial community structure. They discovered that data from samples collected in the natural environment across a multitude of gradients (e.g., pH, temperature, atmospheric pressure) separated primarily based on whether the samples originated from saline or non-saline environments—despite the substantial technical differences between studies. Fascinatingly, when these same data were combined with samples collected from vertebrate guts, the primary variation in the data was explained by whether the samples were environmental or host associated [1], implying that an extremely high degree of specialization has occurred in the microbial communities of vertebrate guts (which is particularly interesting given the difference in evolutionary time that environmental microbial communities have had to specialize relative to the time that vertebrates have existed). While this meta-analysis did not employ a reference set of the type discussed here, it has itself become a de facto reference set that has subsequently been employed for comparison with numerous other studies [48–50].

More recently, a re-evaluation of a longitudinal study aimed at exploring succession in microbial communities within an infant (for the original study, see [24]) was performed using the HMP as context [31]. While the original work showed a distinct increase in the diversity of the infant’s fecal community through the first few years of life, putting its results in context immediately clarified the trajectory of succession by showing that the microbiome moved from resembling a vaginal community (which makes sense given the mode of birth, see [51] for a study on the effects of delivery mode on the infant microbiome) to resembling a fecal community. Visualizing longitudinal microbiome studies as animations (see [52] for a movie of the re-evaluation of the aforementioned infant longitudinal data), particularly in the context of a reference, has been so useful that the ability was recently added into EMPeror [53], a common visualization tool for ordination plots generated from microbiome data.

Meta-analyses are becoming more widespread as computational power increases, sometimes employing past studies that were not intended as reference sets in that new role. Moeller et al. [29] reused the Global Gut [28] data to paint a compelling picture of the coevolution of hominids and their gut communities, highlighting a departure that humans have appeared to take with respect to our closest ancestors. The data suggest that the rate of change in the human microbiome is significantly higher since divergence with chimpanzee, particularly in US adults, including a significant decrease in alpha diversity. The motivation to reuse the Global Gut data was access to samples collected from hunter-gatherer groups as well as western adults, enabling the researchers to test the hypothesis that hunter-gatherer groups are more similar from a microbial perspective to our closest ancestors potentially due to the dramatic dietary differences that exist between these groups and western populations. However, the sample size for any given age group and population combination (e.g., infant Malawians) within Global Gut was relatively small, so it would be interesting to revisit this and see what the pattern of coevolution is against a reference that contains a larger number of samples for different age groups.

### Contributions of the American Gut Project

The American Gut Project set out to build a comprehensive open-source and open-access microbiome 16S rRNA reference dataset for the scientific community to use. It relies on a crowd-funding model that allows for broad reach across the US population and is set up so that virtually anyone can participate (with the exception of convicted felons and children younger than 6 weeks old). Individuals can elect to receive a collection kit in exchange for a contribution to the project. Though the sample population is not free from bias (being shifted toward older Caucasians interested in their own health), the variability encompassed by the project vastly exceeds that of the HMP [36]. In addition, the project has recently expanded internationally to the UK and Australia to reduce participant overhead for shipping samples (although, to minimize the introduction of technical variability, all samples are extracted at one site, UC San Diego). All participants in the project are consented under protocol #141853 approved by the University of California San Diego’s Human Research Protection Program (HRPP); the protocol specifies that all non-identifying data collected will be deposited into the public domain. Each participant is presented with a HRPP-approved questionnaire that covers diet, lifestyle, and health history, including a NIH-validated food frequency questionnaire [54]. The infrastructure to support electronic consent, questionnaires, localization for international portals, and management of over 22,000 bar-coded samples has opened the doors for external researchers and the general public alike to perform their own experiments using the framework of the American Gut Project.

The American Gut Project is a subset of the Earth Microbiome Project (EMP) [19], which has been instrumental in advocating for adherence to the standards of the Genomics Standards Consortium, including minimum information about a marker gene sequence (MIMARKS) [55]—a suite of standards defining variables to be collected within a marker gene survey for virtually any environment imaginable. The EMP and American Gut also follow published sequencing protocols [56] that aim to normalize technical bias for microbiome studies and employ the Biological Observation Matrix (BIOM) [44] specification as a standard and computationally efficient means to represent the resulting large, sparse-omics datasets and their sample and observation metadata. All data are de-identified and deposited into the public domain as quickly as possible via the European Bioinformatics Institute (EBI), which is part of the International Nucleotide Sequence Database Consortium (INSDC). American Gut has taken a further step by providing executable IPython [57] Notebooks allow others to reproduce and modify the analyses being performed on the data. All code for the project is hosted on Github in the “biocore” organization and is available under the BSD license, and all code and binaries used by the project are open-source.

Although the American Gut is useful, by design it is not intended to provide an unbiased population but rather to harness crowd funding and public enthusiasm to uncover the range of extant microbiomes. Given this fact, many questions could be best addressed by instead adding microbiome components to existing carefully designed cohorts such as NHANES [58], the Nurses’ Health Study [59], and TwinsUK [60]. Relevant areas of inquiry include relating the microbiome to heart disease, cancer, stroke, cognitive abilities, and host genetics, as well as leveraging new avenues to assess sources of technical variation. These studies offer the unique potential to build off of their already well-characterized populations.

## Conclusions

Research is never performed in isolation. It is built upon the foundations laid by prior knowledge and evaluated in the context of present knowledge. However, if data are not collected with a view toward integration, or if rich reference points do not exist, research is effectively performed in a vacuum. These are some of the challenges that a common reference can help to address, and the American Gut is a widely collaborative, carefully structured project that aims to provide such a reference. The establishment of a comprehensive reference encourages widespread use of standard protocols, since normalization of technical variation is essential when comparing results to the reference and assessing the significance of a study against the background population. Application of context-aware study designs that adhere to community-accepted standards used by references like the American Gut should minimize the time until microbiome research findings become medically actionable.

## Abbreviations

BIOM: Biological Observation Matrix
EBI: European Bioinformatics Institute
EMP: Earth Microbiome Project
HMP: Human Microbiome Project
HRPP: Human Research Protection Program
INSDC: International Nucleotide Sequence Database Consortium
MBQC: Microbiome Quality Control Project
MD-index: microbial dysbiosis index
MIMARKS: minimum information about a marker gene sequence
OTU: operational taxonomic unit
